# Comparison of Pharmacokinetics of the GalNAc-Conjugated Antisense Oligonucleotide GSK3389404 in Participants with Chronic Hepatitis B Infection across the Asia-Pacific Region

**DOI:** 10.1128/aac.00900-22

**Published:** 2022-12-12

**Authors:** Kelong Han, Hiroshi Ito, Robert Elston, Jennifer Cremer, Steve Hood, Melanie Paff, Dickens Theodore

**Affiliations:** a GSK, Collegeville, Pennsylvania, USA; b GSK K.K., Tokyo, Japan; c GSK, Stevenage, United Kingdom; d GSK, Durham, North Carolina, USA

**Keywords:** Asia-Pacific, GSK3389404, antisense oligonucleotide, chronic hepatitis B virus infection, pharmacokinetics

## Abstract

GSK3389404, an N-acetyl galactosamine-conjugated antisense oligonucleotide (ASO), was in clinical development for chronic hepatitis B (CHB) treatment. Few studies have examined ASOs in Asian participants. In this analysis, the plasma pharmacokinetics (PK) of GSK3389404 were characterized and compared in patients with CHB across the Asia-Pacific region (N = 64), including mainland China (*n* = 16), Hong Kong (*n* = 8), Japan (*n* = 21), South Korea (*n* = 12), Singapore (*n* = 4), and the Philippines (*n* = 3), from a phase 2a, multicenter, randomized, double-blind, placebo-controlled study (NCT03020745). Hepatitis B(e) antigen-positive and -negative patients (on or not on stable nucleos[t]ide regimens) received single (30 mg or 120 mg) or multiple (30 mg, 60 mg, or 120 mg weekly or 120 mg biweekly) subcutaneous GSK3389404 injections. The plasma concentrations were measured on day 1 in all cohorts as well as on days 29 and 57 in the multiple-dose cohorts. The GSK3389404 plasma PK were similar to those reported in a previous study in non-Asian healthy participants with a median time to peak concentration (t_max_) of 1 to 4 h postdose, a mean half-life of 3 to 5 h across cohorts, and no accumulation following repeat dosing. The GSK3389404 plasma t_max_ and half-life values were dose-independent. The increase in the plasma peak concentration (C_max_) and the area under the concentration versus time curve (AUC) was dose-proportional from 60 to 120 mg and greater than dose-proportional from 30 to 60 or 120 mg. The GSK3389404 plasma concentration versus time profiles, half-life, t_max_, C_max_, and AUC values were all comparable across the Asia-Pacific populations. Given the similarity of the PK among ASOs, this analysis suggests that the PK from any Asia-Pacific population may be used to guide ASO dose selection in the Asia-Pacific region.

## INTRODUCTION

The global prevalence of chronic hepatitis B virus (HBV) infection was estimated to be 296 million in 2019, with approximately 1.5 million new infections being reported each year (1). A disproportionate burden of chronic HBV infections have been reported among Asians and Pacific Islanders, with 18 million cases in southeast Asia alone (1) and an estimated 84 million cases in China in 2018 (2). In 2019, chronic HBV infections accounted for 820,000 deaths, primarily from cirrhosis and hepatocellular carcinoma (1). Two nucleos(t)ide analogues (NAs), tenofovir and entecavir, as well as the immune modulator pegylated interferon are recommended by the World Health Organization for the treatment of chronic HBV infection (3). NAs are effective at suppressing HBV DNA levels, but they are not capable of curing the disease (4), and the use of pegylated interferon is limited due to its variable effects and poor safety profile (3, 5). This highlightings the need for novel treatment options.

GSK3389404 is the N-acetyl galactosamine (GalNAc)-conjugated version of bepirovirsen (GSK3228836). Bepirovirsen is a 20-mer antisense oligonucleotide (ASO) against a shared region that is present in all HBV mRNAs and pregenomic RNA, and it is designed to reduce HBV DNA and the expression of the HBV surface antigen (HBsAg). Bepirovirsen is currently being investigated in clinical trials for the treatment of patients with chronic HBV infections (6). GSK3389404 was developed in parallel to provide enhanced delivery to hepatocytes (7, 8). In a preclinical transgenic mouse model of HBV infection, GSK3389404 mediated robust dose-dependent reduction in HBsAg (9).

The first-in-human study of GSK3389404 (NCT02647281) assessed plasma pharmacokinetics (PK) and safety in healthy, predominantly Caucasian participants following single (10 mg, 30 mg, 60 mg, and 120 mg) and multiple (30 mg, 60 mg, and 120 mg weekly for 4 weeks) subcutaneous injections of GSK3389404 (10). The study showed that in healthy non-Asian participants, GSK3389404 is rapidly absorbed following a subcutaneous injection, with the time to the maximum observed plasma concentration (t_max_) ranging from 1 to 4 h postdose across cohorts and a mean plasma half-life (both the arithmetic mean and the geometric mean) of 3 to 6 h across all dose levels (10). Overall, the GSK3389404 plasma area under the concentration versus time curve (AUC) and the maximum observed plasma concentration (C_max_) increased in a dose-proportional manner. The GSK3389404 plasma PK profiles and parameters were comparable between participants receiving single and multiple doses as well as between the first and fourth weekly dose, indicating no plasma accumulation following repeat dosing (10). Unchanged GSK3389404 detected in the urine represented <0.1% of the administered dose (10).

No additional drug-related material was detected in the urine or plasma (10). No safety concerns were identified with GSK3389404 over the dose range evaluated, thereby supporting a Phase 2a study in participants with chronic HBV infections.

A phase 2a study (NCT03020745) was conducted in participants with chronic HBV infections in Asia, including mainland China, Hong Kong, Japan, South Korea, the Philippines, and Singapore, to assess the safety, tolerability, and PK of single and multiple doses of GSK3389404 in participants with chronic HBV infections (11). GSK3389404 demonstrated a favorable safety profile. Although target engagement with dose-dependent reductions in HBsAg levels was observed, no effective dosing regimen was identified (11).

The objectives of this analysis were to characterize the plasma PK of single and multiple GSK3389404 doses in Asian participants with chronic HBV infections from study NCT03020745 and to compare the GSK3389404 plasma PK across Asian-Pacific populations from mainland China, Hong Kong, Japan, South Korea, Singapore, and the Philippines.

## RESULTS

### Participants.

A total of 65 participants received at least one dose of GSK3389404 (11), 64 of whom had evaluable plasma PK data and were included in this analysis. 1 participant from China in the 120 mg biweekly treatment arm in Part 2 (main study) was excluded from the analysis due to a nonzero GSK3389404 plasma concentration prior to the first dose, and this participant only completed day 1 of the study.

The 64 participants included in this analysis were from mainland China (*n* = 16), Hong Kong (*n* = 8), Japan (*n* = 21), South Korea (*n* = 12), Singapore (*n* = 4), and the Philippines (*n* = 3). The baseline demographics and clinical characteristics of the analysis population were similar across populations (Table 1). The mean age was 40.3 to 51.3 years, and most of participants (57.1% to 87.5%) were male. The mean body weight was 61.3 to 69.9 kg, and the mean body mass index was 22.1 to 24.3 kg/m^2^ across the populations.

**TABLE 1:** Baseline characteristics

Characteristic^*a*^	Mainland China (*n* = 16)	Hong Kong (*n* = 8)	Japan (*n* = 21)	South Korea (*n* = 12)	The Philippines (*n* = 3)	Singapore (*n* = 4)
Full PK profile^*b*^, *n*	0	6	18	3	0	0
Receiving NA treatment, *n* (%)	16 (100%)	4 (50%)	21 (100%)	11 (91.7%)	3 (100%)	4 (100%)
Mean (95% CI) age, yrs	47.8 (41.9, 53.7)	48.6 (35.2, 60.3)	51.3 (47.6, 54.2)	49.8 (43.5, 54.8)	40.3 (26.2, 57.6)	46.3 (36.8, 56.1)
Male, *n* / %	13 / 81.2%	7 / 87.5%	12 / 57.1%	9 / 75.0%	2 / 66.7%	3 / 75.0%
HBeAg negative, *n* / %	9 / 56.2%	7 / 87.5%	20 / 95.2%	8 / 66.7%	2 / 66.7%	3 / 75.0%
Mean (95% CI) HBsAg, log_10_IU/mL	2.7 (2.5, 3.0)	3.0 (2.4, 3.5)	2.9 (2.7, 3.1)	2.9 (2.7, 3.1)	3.4 (3.1, 3.8)	2.6 (2.2, 3.1)
Mean (95% CI) wt, kg	69.7 (65.1, 74.3)	64.4 (60.3, 68.2)	61.3 (56.2, 65.1)	65.3 (59.7, 70.1)	62.3 (48.1, 78.4)	69.9 (52.4, 88.5)
Mean (95% CI) BMI, kg/m^2^	23.6 (22.6, 24.6)	22.3 (21.2, 23.4)	22.4 (21.1, 23.5)	23.7 (22.3, 25.0)	22.1 (18.9, 25.7)	24.3 (21.1, 27.6)
Mean (95% CI) ALT, U/L	18.6 (12.8, 24.3)	19.8 (16.5, 22.5)	20.8 (15.5, 23.0)	22.6 (15.2, 26.0)	23.3 (19.2, 27.9)	23.5 (18.6, 28.7)
ALT >ULN^*c*^, *n* / %	2 / 12.5%	0	0	1 / 8.3%	0	0

a ALT, alanine aminotransferase; BMI, body mass index; CI, confidence interval; HBeAg, hepatitis B(e) antigen; HBsAg, hepatitis B surface antigen; NA, nucleos(t)ide analogue; PK, pharmacokinetics; ULN, upper limit of normal; yrs, years; wt, weight.

b A full PK profile consisted of plasma samples that were regularly collected for at least 8 h postdose.

c No participant had ALT >2× ULN at the baseline.

No renal safety signal, including individual and mean changes from the baseline, was identified in participants receiving GSK3389404 during the study. There was no confirmed creatinine change from the baseline of >0.3 mg/dL. The mean glomerular filtration rate (mL/min/1.73 m^2^) values at the baseline ranged from 100.0 to 110.7 across the cohorts in Part 1, from 92.2 to 100.4 across the cohorts in Part 2 (main study), and from 79.7 to 92.2 in Part 2 (Japanese substudy). The majority of the participants across Part 1 and Part 2 were of East Asian (*n* = 31 [48.4%]) and Japanese heritage (*n* = 21 [32.8%]), followed by Southeast Asian (*n* = 11 [17.2%]) and Mixed East Asian/Southeast Asian heritage (*n* = 1 [1.6%]).

### GSK3389404 plasma PK profiles and PK parameters.

A reduced PK profile (predose as well as 1, 2, and 3 h postdose) was available for all participants, whereas full PK profiles (plasma samples collected for at least 8 h postdose) were available in 27 participants from Hong Kong (*n* = 6, Part 1), Japan (*n* = 18, Part 2 of the Japanese substudy), and South Korea (*n* = 3, Part 1). In the 120 mg biweekly (once every 2 weeks) treatment arm in Part 2 (main study, reduced PK profile), 1 participant had a 1,000-fold higher GSK3389404 plasma concentration, compared with the other participants who received 120 mg biweekly. Therefore, this participant was excluded as an outlier.

**FIG 1:**
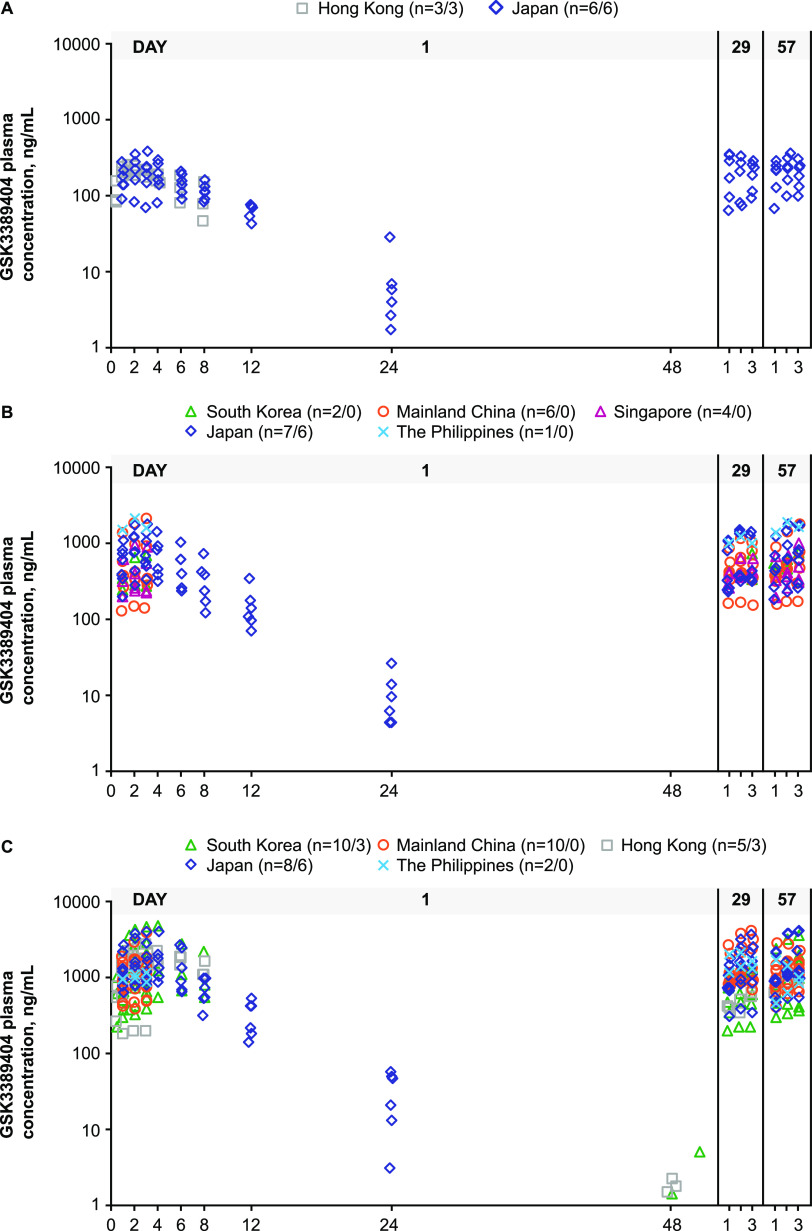
GSK3389404 plasma concentration versus time profiles after the first dose of (A) 30 mg, (B) 60 mg, and (C) 120 mg across the Asia-Pacific region. The 120 mg weekly and 120 mg biweekly cohorts were pooled together, given that the PK profiles were after the first dose. Intensive PK profiles and reduced PK profiles (predose as well as 1, 2, and 3 hours postdose) are both displayed. The N numbers indicate the total number of participants or the number of participants with an intensive PK profile. PK, pharmacokinetics.

The GSK3389404 plasma concentration versus time profiles at each dose level, following single or multiple doses, are displayed in Fig. 1. The PK parameters estimated with full PK profiles are summarized in Tables 2 and 3. The GSK3389404 was rapidly absorbed following subcutaneous administration, with a median time to C_max_ (t_max_) of 1 to 4 h across cohorts (Table 2). The geometric mean of the plasma half-life (t_1/2_) was 3 to 5 h across cohorts.

**TABLE 2:** GSK3389404 plasma PK parameters following the first dose in participants with full PK profiles^*a*^ across the Asia-Pacific region

	Hong Kong		Japan			South Korea
Parameter^*b*^	30 mg GSK3389404 single dose (*n* = 3)	120 mg GSK3389404 single dose (*n* = 3)	30 mg GSK3389404 weekly (*n* = 6)	60 mg GSK3389404 weekly (*n* = 6)	120 mg GSK3389404 weekly/biweekly (*n* = 6)	120 mg GSK3389404 single dose (*n* = 3)
C_max_, ng/mL	227 (205, 255)	2,058 (1,750, 2,750)	214 (90.1, 386)	865 (520, 1,500)	1,795 (1,020, 4,030)	1,718 (670, 4,760)
AUC_0–8_, h·μg/mL	1.24 (1.12, 1.45)	11.94 (10.35, 14.90)	1.23 (0.63, 2.03)	4.29 (2.75, 8.57)	9.59 (5.64, 21.98)	9.57 (4.04, 25.59)
t_1/2_, h	3.12 (2.17, 4.48)^*c*^	4.27 (4.13, 4.41)^*c*^	3.90 (2.64, 10.52)	3.24 (2.24, 4.36)	3.50 (1.88, 5.86)	4.40^*d*^
t_max_, h	1.55 (1.33, 2.00)	3.23 (1.93, 5.93)	2.12 (0.90, 3.98)	3.21 (1.98, 5.98)	3.66 (2.02, 6.03)	4.16 (3.00, 6.00)

a A full PK profile consisted of plasma samples that were regularly collected for at least 8 h postdose.

b The geometric mean (range) is shown, except for t_max_, which is shown as the median (range). AUC_0–8_, area under the plasma concentration versus time curve from time zero (predose) to 8 h postdose; C_max_, maximum observed plasma concentration; PK, pharmacokinetics; t_1/2_, plasma half-life; t_max_, time to C_max_.

c *n* = 2.

d *n* = 1.

**TABLE 3:** Derived GSK3389404 PK parameters following the first dose (Japanese substudy)^*a*^

Parameter^*b*^	30 mg weekly (*n* = 6)	60 mg weekly (*n* = 6)	120 mg weekly/biweekly (*n* = 6)
AUC_(0-inf)_ (h·μg/mL)	2.03 (1.65, 2.75) [1.650, 2.458]	5.99 (3.91, 10.2) [3.909, 8.692]	13.4 (9.28, 24.0) [9.147, 20.36]
AUC_(0-8)_ (h·μg/mL)	1.23 (0.633, 2.03) [0.812, 1.867]	4.29 (2.75, 8.57) [2.719, 6.776]	9.59 (5.64, 22.0) [5.634, 16.33]
AUC_(0-24)_ (h·μg/mL)	1.93 (1.53, 2.74) [1.545, 2.409]	5.92 (3.82, 10.2) [4.094, 8.555]	13.1 (8.87, 24.0) [8.738, 19.78]
C_max_ (ng/mL)	214 (90.1, 386) [125.4, 364.5]	865 (520, 1,500) [570.0, 1,312]	1,800 (1,020, 4,030) [1,070, 3,012]
C_24_ (ng/mL)	5.25 (1.73, 28.4) [1.899, 14.52]	8.86 (4.47, 27.0) [4.208, 18.65]	22.3 (3.10, 58.1) [6.845, 72.43]
CL/F (L/h)	14.8 (10.9, 18.2) [12.30, 17.78]	10.0 (5.86, 15.3) [6.993, 14.35]	8.96 (5.00, 12.9) [6.034, 13.31]
t_1/2_ (h)	3.90 (2.64, 10.52) [2.275, 6.695]	3.24 (2.24, 4.36) [2.479, 4.239]	3.50 (1.88, 5.86) [2.297, 5.336]
t_max_ (h)	2.12 (0.90, 3.98)	3.21 (1.98, 5.98)	3.66 (2.02, 6.03)

a Source: (11).

b The geometric mean (range) [geometric 95% CI] is displayed, except for t_max_, which is shown as the median (range). AUC_0–inf_, area under the plasma concentration versus time curve from time zero (predose) extrapolated to infinity; AUC_(0–8)_, area under the plasma concentration versus time curve from time zero (predose) to 8 h; AUC_(0–24)_, area under the plasma concentration versus time curve from time zero (predose) to 24 h; C_24_, plasma concentration at 24 h postdose; CI, confidence interval; CL/F, apparent subcutaneous plasma clearance; C_max_, maximum observed plasma concentration; PK, pharmacokinetics; t_1/2_, plasma half-life; t_max_, time to the maximum observed plasma concentration.

The GSK3389404 plasma concentration was detectable at 24 h post dose at all dose levels, but it was only detectable at 48 h post dose at the highest dose level of 120 mg (Fig. 1C), and it was only detectable in 5 out of the 6 participants who received 120 mg and provided a 48 h PK sample. The median and maximum plasma concentrations at 48 h postdose were 1.67 ng/mL and 5.09 ng/mL, respectively, close to those of the assay lower limit of quantification (LLOQ) of 1 ng/mL. Following repeat dosing, the postdose plasma PK profiles were similar on days 1, 29 (week 4), and 57 (week 8), and the plasma PK profiles were similar between the 120 mg weekly and 120 mg biweekly regimens, indicating no accumulation in plasma concentration following repeat dosing (Fig. 1).

**FIG 2:**
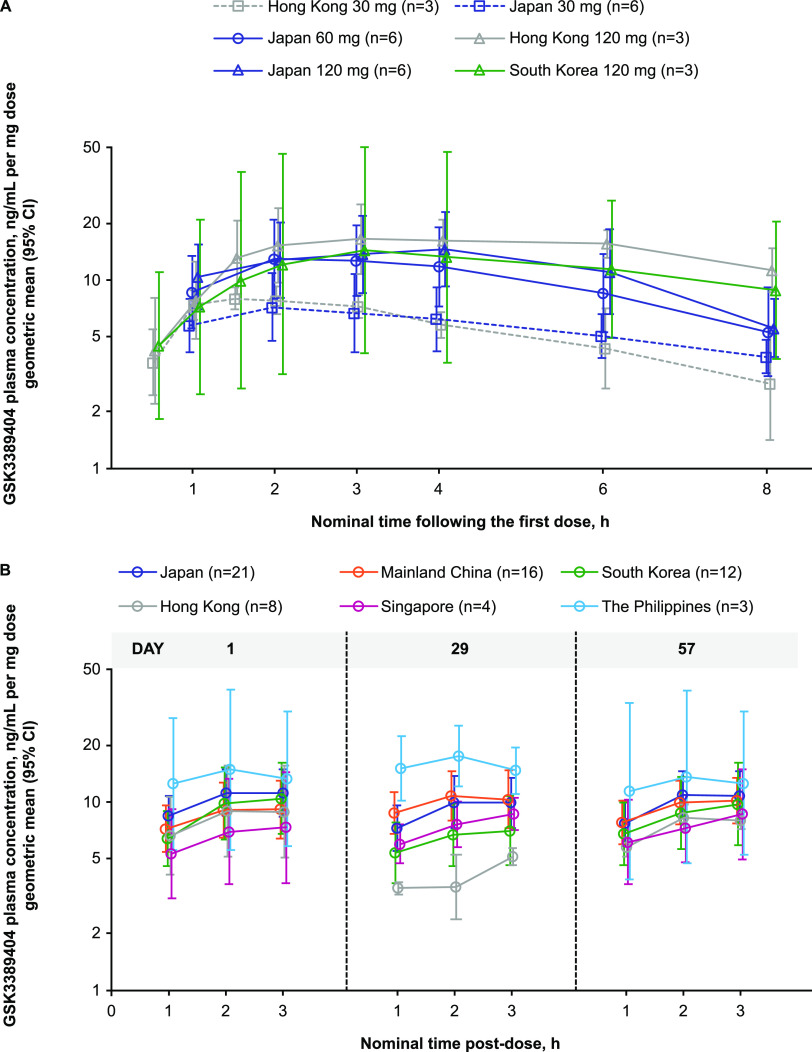
GSK3389404 dose-normalized plasma concentration-versus-time profiles across the Asia-Pacific region in (A) participants with full PK profiles (consisting of plasma samples that were regularly collected for at least 8 hours post dose) and (B) participants with reduced PK profiles (consisting of plasma samples that were regularly collected up to 1, 2, and 3 hours post dose). CI, confidence interval; PK, pharmacokinetics.

The GSK3389404 plasma t_max_ and t_1/2_ were dose-independent (Tables 2 and 3). The increase in the plasma C_max_ and AUC from time zero (predose) to 8 h (AUC_0–8_) was dose-proportional from 60 to 120 mg and greater than dose-proportional from 30 to 60 or 120 mg (Table S1). The dose-normalized plasma PK profiles were similar between 60 mg and 120 mg but were lower for 30 mg (Fig. 2A).

### Comparison of GSK3389404 plasma PK across Asia-Pacific populations.

**FIG 3:**
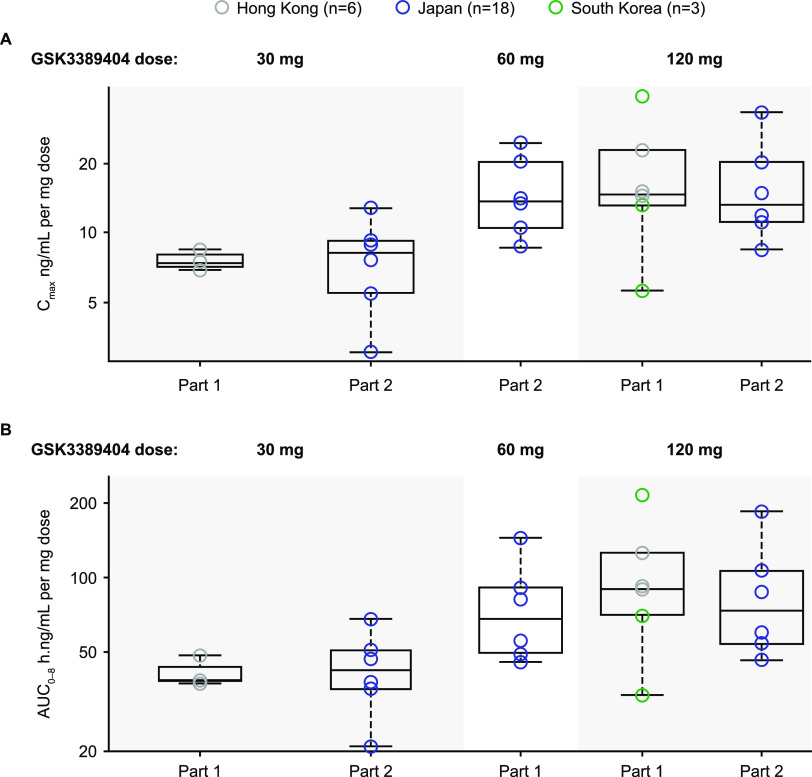
GSK3389404 dose-normalized plasma (A) C_max_ and (B) AUC_0–8_ following the first dose in participants with full PK profiles (consisting of plasma samples that were regularly collected for at least 8 hours post dose) across the Asia-Pacific region. AUC_0–8_, area under the plasma concentration-versus-time curve from time zero (predose) to 8 hours post dose. C_max_, maximum observed plasma concentration; PK, pharmacokinetics.

The GSK3389404 plasma concentration-versus-time profiles were similar across the populations from mainland China, Hong Kong, Japan, South Korea, Singapore, and the Philippines at the same dose level, following single or multiple doses (Fig. 1 and 2). The GSK3389404 plasma C_max_, AUC_0–8_, t_max_, and t_1/2_ were similar across the populations from Hong Kong, Japan, and South Korea at the same dose level (Table 2; Fig. 3).

## DISCUSSION

This analysis characterized the plasma PK of single and multiple doses of GSK3389404 in participants with chronic HBV infections and compared the GSK3389404 plasma PK across Asian-Pacific populations from mainland China, Hong Kong, Japan, South Korea, the Philippines, and Singapore. The data showed that the GSK3389404 plasma concentration versus time profiles, PK parameters, and exposure were similar across the Asian-Pacific populations assessed.

In studies enrolling patients with the same ethnic background, PK, efficacy, and safety profiles are expected to be similar, although this has not yet been established (12). An evaluation of the PK and safety profiles of four different compounds, with various mechanisms of action and characteristics, in East Asian populations (Japan, China, South Korea, and Taiwan) revealed no distinct differences across populations (12). This is consistent with the results given in previous reports (13), and the authors concluded that these results may support the mutual use of clinical data among East Asian countries (12). Similarly, a recent review that assessed the value of separate ethnic sensitivity studies (ESS) in Asian populations found the value of ESS to be limited for drugs considered to be ethnically insensitive (e.g., therapeutic biologics, drugs with no systemic exposure, drugs predominantly metabolised by cytochrome P450 [CYP] enzymes with known polymorphisms that are of no functional relevance or with functionally polymorphic CYP enzymes) (14). This led the authors to propose a new global drug development model for such ethnically insensitive drugs, in which relevant safety, PK, and pharmacogenetic data from an original phase 1 study could be extrapolated to Asian populations in order to streamline their inclusion into phase 2/3 trials without requiring an ESS (14). This is in line with the International Conference on Harmonisation E5 guideline, which recommends no bridging studies in cases in which the drug is ethnically insensitive and extrinsic factors (e.g., medical practice, clinical trial conduct) are similar between regions (15, 16).

There is a paucity of published data assessing the PK of ASOs across different countries within Asia or between Asians and non-Asians, which may promulgate the continued requirement of ESS. Differences in PK across the Asian-Pacific populations were not expected, and this is confirmed by the results of this analysis. GSK3389404 metabolism and elimination do not involve hepatic enzymes and transporters. The elimination of GSK3389404 is expected to be via metabolism in the tissue and renal elimination of shortened chain fragments, based on studies with other ASOs (17). A model compound of 2′-O-(2-methoxyethyl)-modified ASO was shown to be neither a substrate nor an inhibitor of organic anion transporter (OAT)1, OAT3, organic cation transporter (OCT)1, and OCT2 (18). The primary route of elimination of GSK3389404 is expected to be via metabolism by endogenous endonucleases, and there is no known pharmacogenomic difference in endogenous endonucleases between Asian-Pacific populations. In Fig. 2B, the variability across populations seems to be less on days 1 and 57 than on day 29, and this result is likely due to random bias, given the small sample size.

The GSK3389404 plasma PK in participants with chronic HBV infections in this study (GSK study 205670) are consistent with those reported in a previous study in non-Asian healthy participants (GSK study 202007) (10). The median plasma t_max_ was 1 to 4 h, the geometric mean plasma t_1/2_ was 3 to 5 h across cohorts, and both the plasma t_max_ and t_1/2_ were dose-independent, consistent with those of the non-Asian healthy participants (10). The geometric mean ratios of the dose-normalized plasma C_max_ and AUC_0–inf_ between studies 202007 (non-Asian) and 205670 (Asian) are 0.737 and 0.734, respectively. A slightly higher exposure in Asian participants than non-Asian participants is expected, since the Asian participants received a higher mg/kg dose due to their lower body weights. However, the difference in plasma exposure between studies 202007 and 205670 is unlikely to be relevant. First, this difference of 26 to 27% is relatively small, compared with the overall variability in the plasma PK. Furthermore, the dose-normalized plasma C_max_ and AUC_0–inf_ values are within the same ranges in these two studies. The C_max_ range was 3.98 to 26.40 ng/mL/mg in study 202007 and 3.00 to 39.67 ng/mL/mg in study 205670, and the AUC_0–inf_ range was 29.60 to 236.67 h·ng/mL/mg in study 202007 and 43.54 to 200.00 h·ng/mL/mg in study 205670. Finally, plasma exposure only accounts for a minor portion of the total dose, and this small difference is unlikely to result in a clinically relevant difference.

The increase in the GSK3389404 plasma C_max_ and AUC values was dose-proportional at higher the dose levels (from 60 mg to 120 mg) and greater than dose-proportional from the low dose level to the higher dose levels (from 30 mg to 60 mg and from 30 mg to 120 mg), which was consistent with the results seen with bepirovirsen (unconjugated ASO) in healthy non-Asian participants (19) as well as in animal studies of both GSK3389404 and bepirovirsen (unpublished data). Geary et al. speculate that this is due to a low capacity off-target binding site that is saturated once the doses rise (20).

The observed GSK3389404 plasma PK profiles were similar to those of bepirovirsen (19) and other ASOs (21–23) in that they featured a rapid absorption phase postdose that was followed by an initial rapid distribution phase. Given that ASOs and their GalNAc-conjugated versions were shown to have similar plasma elimination half-lives (23) and that GSK3389404 and bepirovirsen share the same core structure, it is reasonable to expect that the plasma elimination half-life of GSK3389404 should be around 3 weeks, which would be similar to that observed with bepirovirsen at dose levels between 150 mg and 450 mg (19). However, the enhanced hepatocyte delivery offered by GalNAc conjugation allowed for the low dose levels of 30 to 120 mg to be studied for GSK3389404. This is likely the reason that GSK3389404 plasma concentration was only detectable for up to 2 days postdose, and the slower elimination phase likely fell below the LLOQ. The characterization of the plasma t_1/2_ is important because it represents the elimination half-life in tissues, especially the liver. Given that the hepatic concentration of GSK3389404 is approximately 5,000 times higher than that in the plasma (24), the hepatic concentrations may remain above the therapeutic level after the GSK3389404 plasma concentration falls below the LLOQ.

This analysis has several limitations. The PK sampling schedules of the full PK profiles in Part 1 and Part 2 of the Japanese substudy were different. In Part 1, the 12 h and 24 h PK samples were not collected due to operational constraints. As a result, the 0 to 24 h PK profiles, AUC from time zero (predose) to 24 h postdose (AUC_0–24_), and the AUC from time zero (predose) extrapolated to infinite time (AUC_0–inf_) could not be compared in participants with full PK profiles between Part 1 and Part 2 of the Japanese substudy. This limited the comparison of the PK profiles and the AUC to 0 to 24 h PK profiles (Fig. 2) and AUC_0–8_ (Table 2). Furthermore, full PK profiles were not available in Part 2 of the main study due to operational constraints, and instead, reduced PK profiles (up to 3 h postdose) were collected, but these could only be used for comparisons of PK profiles (Fig. 1 and 2) and do not allow for PK parameter estimation. As Part 2 of the main study contained all of the participants enrolled in mainland China, Singapore, and the Philippines, the full PK profiles and PK parameters could only be compared between participants from Hong Kong, South Korea, and Japan (Fig. 2A; Table 2).

In conclusion, the GSK3389404 plasma PK were similar to those observed in the previous study in non-Asian healthy participants and with other ASOs (10, 21–23). The median plasma t_max_ was 1 to 4 h postdose, and the mean plasma half-life was 3 to 5 h across cohorts. No plasma accumulation was observed following repeat dosing. The GSK3389404 plasma PK profiles, half-life, t_max_, AUC, and C_max_ were comparable across the Asia-Pacific populations from mainland China, Hong Kong, Japan, South Korea, Singapore, and the Philippines. Given the similarity of the PK among ASOs, this analysis supports the notion that plasma PK from any Asia-Pacific population may be used to guide the dose selection of ASOs in the Asia-Pacific region.

## MATERIALS AND METHODS

### Study design and population.

The details of the study design and eligibility criteria have been previously published (11). Briefly, this was a phase 2a, multicenter, randomized, double-blind, placebo-controlled study in participants with chronic HBV infections (Table 4). The study was conducted in 2 parts. Part 1 was a single-ascending dose study with three sequential cohorts (GSK3389404 30 mg, 120 mg, and 120 mg) conducted in Hong Kong and South Korea. Part 2 was a multiple-dose, dose-ranging study in 22 sites in the Asia-Pacific region (mainland China, Hong Kong, Japan, South Korea, the Philippines, and Singapore), and it was comprised of a main study and a Japanese substudy. In Part 2, participants who were assigned to an active GSK3389404 treatment received GSK3389404 60 mg weekly, 120 mg weekly, and 120 mg biweekly (once every 2 weeks) for 12 weeks. The Part 2 Japanese substudy contained an additional cohort of 30 mg weekly for 12 weeks to assess PK linearity in the Japanese participants. GSK3389404 was administered via subcutaneous injection.

**TABLE 4:** Study design

Study part	Design	PK sampling schedule	Dose level and population^*a*^
Part 1	Single dose	Full PK profile: predose and 0.5, 1, 1.5, 2, 3, 4, 6, 8, and 48 hours postdose; one sample on days 8 and 30.	30 mg: Hong Kong (3^*b*^). 120 mg: Hong Kong (3^*c*^), South Korea (3^*b*^).
Part 2 main study	Multiple dose for 12 wks	Reduced PK profile on days 1 (after the first dose), 29 (wk 4), and 57 (wk 8): predose and 1, 2, and 3 hours post dose. One sample on day 169 (wk 24).	60 mg weekly (14): mainland China (6), Japan (1), South Korea (2), the Philippines (1), Singapore (4). 120 mg weekly (12): mainland China (5), Japan (2), South Korea (3), the Philippines (2). 120 mg biweekly (12): mainland China (6), Hong Kong (2), South Korea (4).
			
Part 2 Japanese substudy	Multiple dose for 12 wks	Full PK profile on day 1 after the first dose: predose and 1, 2, 3, 4, 6, 8, 12, and 24 hours postdose.	30 mg weekly (6), 60 mg weekly (6), 120 mg weekly (3), 120 mg biweekly (3).
		Reduced PK profile on days 29 (wk 4) and 57 (wk 8): predose, and 1, 2, and 3 hours postdose.	All from Japan.
		One sample on day 169 (wk 24).	

a Number of participants with PK data who were administered GSK3389404 is displayed in parenthesis.

b All participants were on NAs except one participant.

c All participants were on NAs except two participants.

The participants included in this study were adults aged 18 to 70 years with documented chronic HBV infections for at least 6 months prior to screening. Patients were excluded if they had confirmed or suspected hepatocellular carcinoma within 6 months of randomization, a diagnosis or evidence of liver cirrhosis within 12 months of screening, or a positive test for the hepatitis C/D virus or an HIV infection at screening. The participants in Part 1 were either on or not on a stable NA regimen (Table 4), and the participants in Part 2 were on a stable NA regimen, which was defined as no change in the planned NA regimen for ≥6 months prior to screening and no change over the duration of the study. Five participants were receiving an entecavir treatment at baseline.

The study protocol, any amendments, the informed consent, and other information that required preapproval were reviewed and approved by a national, regional, or investigational center ethics committee or an institutional review board (11). The study was conducted in accordance with the International Council on Harmonisation of Technical Requirements for Registration of Pharmaceuticals for Human Use Good Clinical Practice, applicable country-specific requirements, all applicable participant privacy requirements, and the ethical principles outlined in the Declaration of Helsinki. All participants provided informed consent.

### PK assessments.

The PK sampling is summarized in Table 4. In Part 1 (single dose), PK samples were collected predose, at 0.5, 1, 1.5, 2, 3, 4, 6, 8, and 48 h post dose (full PK profile), and at any time on days 8 and 30. In Part 2 (main study; multiple dose), PK samples were collected predose, at 1, 2, and 3 h postdose (reduced PK profile) on days 1, 29 (week 4), and 57 (week 8), and at any time on day 169 (week 24). In Part 2 (Japanese substudy; multiple dose), PK samples were collected predose and at 1, 2, 3, 4, 6, 8, 12, and 24 h postdose (full PK profile) on day 1; predose and 1, 2, and 3 h post dse (reduced PK profile) on days 29 (week 4) and 57 (week 8); and at any time on day 169 (week 24).

In addition, one PK sample was planned to be collected at the time of early termination if applicable. The GSK3389404 plasma concentrations were measured using analytical methods with a lower limit of quantification of 1 ng/mL, as previously described (10).

A noncompartmental PK analysis was performed to estimate the PK parameters, including the plasma half-life (t_1/2_), AUC_0–8_, C_max_, and t_max_ values, based on the full PK profiles from Part 1 and Part 2 (Japanese substudy) and compared across the populations. The reduced concentration versus time profile (predose as well as 1, 2, and 3 h postdose) was available for all participants and was compared across the populations. The PK parameters were summarized using descriptive statistics.

### Data availability.

The data that support the findings of this analysis are available from GSK upon request and approval from www.clinicalstudydatarequest.com. Restrictions apply to the availability of these data.
