# Supplementary material for: Comparison of Pharmacokinetics of the GalNAc-Conjugated Antisense Oligonucleotide GSK3389404 in Participants with Chronic Hepatitis B Infection across the Asia-Pacific Region
Source: Antimicrob Agents Chemother. 2022 Dec 12;67(1):e00900-22. doi: 10.1128/aac.00900-22 (PMC9872700; doi:10.1128/aac.00900-22)
Supplement: Supplemental file 1 — Supplemental material. Download aac.00900-22-s0001.pdf, PDF file, 0.1 MB [file aac.00900-22-s0001.pdf]

SUPPLEMENTARY MATERIALS

Supplementary Table S1. GSK3389404 dose proportionality assessment by ANOVA, following a single dose

Study part	Dose-normalised Parameter	Test Dose, mg	Test n	Reference Dose, mg	Reference n	Ratio (90% CI)
**Part 1**	**$AUC_{0–8}$, h·ng/mL/mg**	120	6	30	3	2.16 (1.07, 4.35)
	**C_{max}, ng/mL/mg**	120	6	30	3	2.07 (0.99, 4.34)
**Part 2 Japanese sub-study**	**$AUC_{0–8}$, h·ng/mL/mg**	120	6	30	6	1.95 (1.24, 3.07)
		120	6	60	6	1.12 (0.71, 1.76)
		60	6	30	6	1.74 (1.11, 2.75)
	**$AUC_{0–24}$, h·ng/mL/mg**	120	6	30	6	1.70 (1.22, 2.37)
		120	6	60	6	1.11 (0.80, 1.55)
		60	6	30	6	1.53 (1.10, 2.13)
	**C_{max}, ng/mL/mg**	120	6	30	6	2.10 (1.31, 3.37)
		120	6	60	6	1.04 (0.65, 1.67)
		60	6	30	6	2.02 (1.26, 3.25)

ANOVA, analysis of variance; $AUC_{0–8/24}$, area under the plasma concentration-versus-time curve from time zero (pre-dose) to 8/24 hours post dose; C_{max}, maximum observed plasma concentration.
